# Risk Factors for Central and Lateral Lymph Node Metastases in Patients With Papillary Thyroid Micro-Carcinoma: Retrospective Analysis on 484 Cases

**DOI:** 10.3389/fendo.2021.640565

**Published:** 2021-03-05

**Authors:** Yijie Huang, Ying Yin, Wenyi Zhou

**Affiliations:** Department of General Surgery, Guangdong Provincial People’s Hospital, Guangdong Academy of Medical Sciences, Guangzhou, China

**Keywords:** papillary thyroid carcinoma, micro-carcinoma, lymph node metastasis, thyroidectomy, risk factor

## Abstract

**Background:**

Papillary thyroid micro-carcinoma (PTMC) is defined as a tumor with a larger diameter ≤1 cm which has an indolent course and satisfying prognosis. However, the incidence of lymph node metastasis of PTMC cannot be ignored. The aim of this study was to assess the incidence of lymph node metastasis in PTMC patients, as well as to evaluate the risk factors for both central lymph node metastases (CLNM) and lateral lymph node metastases (LLNM).

**Methods:**

Patients who underwent thyroidectomy from January 2017 to October 2020, and pathologically diagnosed with PTMC were enrolled in our study and their medical records were collected and analyzed.

**Results:**

A total of 484 PTMC patients were included. The incidence of central and lateral lymph node metastasis was 49.6% and 9.1%, respectively. Multivariate analysis demonstrated as independent risk factors for CLNM male sex, age <40 years, largest tumor size ≥5 mm and bilaterality. Extrathyroidal extension, presence of CLNM, number of CLNM ≥5 were strong indicators for LLNM.

**Conclusion:**

The incidence of lymph node metastases in PTMC is non-negligible. The identification of potential risk factors for CLNM and LLNM would help tailor individual surgical interventions for patients with PTMC.

## Introduction

Thyroid cancer (TC) is considered to be responsible for over 500,000 cancer cases reported from all sources globally in 2018 and ranks ninth among malignant diseases (1). It is well-known that thyroid carcinoma occurs more frequently in females than in males. In 2020, the statistics confirmed TC as the fifth most common malignancy in women (2). Since the late 20^th^ century, the incidence of TC has continued to increase, which is probably attributed to major advances in diagnostic techniques and to ultrasonography, in particular (2).

TC can be categorized into three types, namely: 1) differentiated TC, including papillary (PTC), follicular (FTC), and Hürthle cell (HTC); 2) medullary TC; and 3) anaplastic TC (3). Among these, PTC accounts for 80%–90% of TC (3). Given the indolent nature of PTC, most patients could be cured by surgical resection and radioiodine therapy, with a survival rate reaching over 90% over a 10-year follow-up (4). Papillary thyroid micro-carcinoma (PTMC) is defined as small thyroid papillary cancer with lesions ≤1 cm in diameter (5). Though patients with PTMC are often classified into low-risk population due to its favorable prognosis, it is noteworthy that a characteristic of PTC is the tendency for lymph node metastasis.

According to the 2015 version of American Thyroid Association (ATA) guidelines (6), PTMC lacking aggressive characteristics, including clinically uninvolved lymph nodes (cN0), is classified in the low-risk group and routine prophylactic central lymph node dissection (CLND) is not recommended. However, given the interference with the thyroid gland, the detection of lymph node metastasis in the central compartment remains challenging and largely depends on sonographer skill. Researchers have revealed that a substantial proportion of cN0 patients are confirmed to be pathologically node-involved (pN1) (7–9). Thus, ATA guidelines also indicate that, for patients with predictive factors of disease relapse and poor prognosis, prophylactic CLND should be included as part of the diagnostic workup as the information relative to lymph node status may affect tumor staging and improve clinical decision-making (6).

The objective of this study was to assess the incidence of lymph node metastasis in PTMC patients, and to evaluate risk factors for both central and lateral lymph node metastases, in an attempt to provide clinical evidence for tailored individual surgical management.

## Methods

### Patient Selection and Perioperative Evaluation

Patients who underwent thyroidectomy at the General Surgery Department of Guangdong Provincial People’s Hospital from January 2017 to October 2020 and were pathologically diagnosed with papillary thyroid micro-carcinoma were enrolled in this study. Patients were then screened based on the following inclusion and exclusion criteria for subsequent data analysis. Inclusion criteria were as follows: 1) pathologically-diagnosed with papillary thyroid micro-carcinoma, confirmed with postoperative paraffin-embedded sections; 2) complete medical records were available; and 3) subjected to thyroidectomy as well as CLND with or without lateral lymph node dissection (LLND). The exclusion criteria were as follows: 1) age under 18 years; 2) postoperative pathology revealed mixed histologic types of carcinoma; and 3) incomplete medical records.

Preoperative evaluation included physical examination, thyroiditis assessment, thyroid function assessment, and high-resolution ultrasonography of the neck. Ultrasonographic features of malignancy included: solid hypoechoic nodule(s), irregular margins, microcalcifications, taller than wide shape, and evidence of capsule discontinuities. Moreover, fine needle cytology aspiration (FNA) was adopted in cases with nodules ≥5 mm and with intermediate malignancy suspicion.

Blood tests for parathyroid hormone (PTH) were performed on the day after surgery as well as at regular out-patient follow-up visits. Hypoparathyroidism, the most common complication of thyroid surgery, was defined as low PTH level accompanied by decreased total serum calcium levels (10). Temporary hypoparathyroidism referred to a duration of less than 6 months after surgery, whereas permanent hypoparathyroidism indicates a duration of greater than 6 months (10).

### Surgical Approach and Histopathological Characteristics

Surgical procedures mainly included total thyroidectomy (TT) with CLND and lobo-isthmectomy (LH) with CLND for patients with or without preoperative indications of CLNM, while LLND was only performed in cases diagnosed with LLNM before or during surgery. Cases clinically suspected of having involved central lymph nodes were categorized into cN1 group. Frozen section was performed on all patients during surgical intervention, and the malignancy of thyroid nodule was confirmed before CLND either by preoperative FNA or intraoperative frozen section. Likewise, for those with lateral lymph nodes highly suspicious of metastases with features including increase of lymph node diameter, microcalcifications, hypoechogenicity, cystic changes, peripheral vascularity by ultrasonography (11), FNA before surgery or intraoperative frozen section was adopted to confirmed diagnosis of LLNM.

Micro-carcinoma was defined as a maximum tumor diameter less than or equal to 1 cm. As for multifocal tumors, the tumor size was determined as the largest diameter of the primary tumor. Extrathyroidal invasion and vascular embolus were observed by pathological examination. Hashimoto’s thyroiditis (HT) was confirmed with any of the following: positive thyroglobulin-antibodies (Tg-Ab) and/or thyroid peroxidase-antibodies (TPO-Ab), or positive information at pathological examination. Lymph node yield was defined as the number of lymph nodes dissected, whereas the lymph node ratio was calculated as the ratio of metastatic lymph nodes out of the total lymph nodes removed.

### Statistical Analysis

Categorical variables are reported as frequency with a percentage while continuous variables as a mean with standard deviation. SPSS 23.0 software (SPSS, Inc, Chicago, Ill) was employed for statistical analysis. Univariate analysis was used to screen for risk factors of CLNM and LLNM, and a multivariate regression model was adopted for further evaluation. For univariate analysis, p<0.1 was considered statistically significant, while p <0.05 was adopted for multivariate analysis (two-sided).

## Results

### Baseline Characteristics

A total of 484 patients with PTMC who had met the inclusion criteria were enrolled in this study. All patients underwent central compartment lymph node dissection, while lateral cervical lymph node dissection was only performed for those with ultra-sonographic indication for LLNM before surgery.

As summarized in **Table 1**, there were 121 males (25%) and 373 females (75%) included in this study, with an average age of 42.3 ± 11.8 years. Among those patients, LH accompanied with CLND was performed in 207 (42.8%) cases, TT with CLND in 233 (48.1%) cases, and TT with CLND and LLND in 44 (8.9%) cases. Eight (1.7%) patients were incidentally diagnosed with PTMC, whereas for 476 (98.3%) cases patients were indicated by preoperative examinations, among which 82 (16.9%) patients were diagnosed by preoperative FNA.

**Table 1:** Baseline characteristics of 484 patients with papillary thyroid micro-carcinoma.

Variables	N = 484 (%)
Sex	
Male	121 (25)
Female	373 (75)
Age (years) (range)	42.3 ± 11.8 years (18–75)
<40	215 (44.4)
≥40	269 (55.6)
Surgical procedure	
LH+CLND	207 (42.8)
TT+CLND	233 (48.1)
TT+CLND+LLND	44 (9.1)
Histopathological features	
Multifocal tumor	
Yes	57(11.8)
No	427 (88.2)
Bilateral tumor	
Yes	87 (18)
No	397 (82)
LTD (cm) (range)	0.65 ± 0.23 cm (0.06–1)
<0.5	97 (20)
0.5-1	387 (80)
Extrathyroidal invasion	
Yes	102 (21.1)
No	382 (78.9)
Vascular embolus	
Yes	2 (0.4)
No	482 (99.6)
HT	
Yes	168 (34.7)
No	316 (65.3)
CLNM	
Yes	240 (49.6)
No	244 (50.4)
LLNM	
Yes	44 (9.1)
No	440 (90.9)
Central lymph node yield (range)	6.3 ± 4.3 (1–27)
Metastatic lymph node amount (range)	2.5 ± 2.1 (1–13)
Lymph node ratio (range)	0.48 ± 0.3 (0.05–1)
Lateral lymph node yield (range)	22.2 ± 12.6(6–66)
Number of cN0 patients	432 (89.3)
Number of cN1 patients	52 (10.7)
Unexpected CLNM	188 (38.8)
Skip metastasis in LLNM	3 (0.6)
Postoperative hypoparathyroidism	
Yes (temp/perm)	123 (25.4)/5 (1)
No (temp/perm)	361 (74.6)/479 (99)

TT, total thyroidectomy; LH, lobo-isthmectomy; CLND, central compartment lymph node dissection; LLND, lateral lymph node dissection; LTD largest tumor diameter; HT, Hashimoto’s thyroiditis; temp/perm temporary/permanent.

According to the pathological features obtained from paraffin section, the average tumor diameter was 0.65 ± 0.23 cm. The presence of CLNM was confirmed in 240 (49.6%) cases, with a mean lymph node yield of 6.3 ± 4.3. More specifically, the average number of involved central lymph nodes was 2.5 ± 2.1, with an average lymph node ratio of 0.48 ± 0.3. Interestingly, the number of cN0 and cN1 patients were 432 (89.3%) and 52 (10.7%), respectively. CLNM was discovered incidentally in 188 (38.8%) cases and was pathologically confirmed in 52 (10.7%) cN1 patients. The sensitivity of preoperative ultrasonography was 21.7%.

Conversely, the incidence of LLNM was 9.1%, with a mean lymph node yield of 22.2 ± 12.6, ranging from 6 to 66. Moreover, skip metastasis (presence of LLNM without involving central compartment) was observed in three cases (0.6%).

As for postoperative complications, temporary hypoparathyroidism was observed in 123 patients (25.4%), usually presented with tingling or numbness in fingers, toes, or around the mouth. However, the PTH level gradually recovered within 6 months after surgery in most cases, with five patients (1%) experienced permanent hypoparathyroidism.

### Univariate and Multivariate Analyses of Risk Factors for Central Lymph Node Metastases

**Figure 1:**
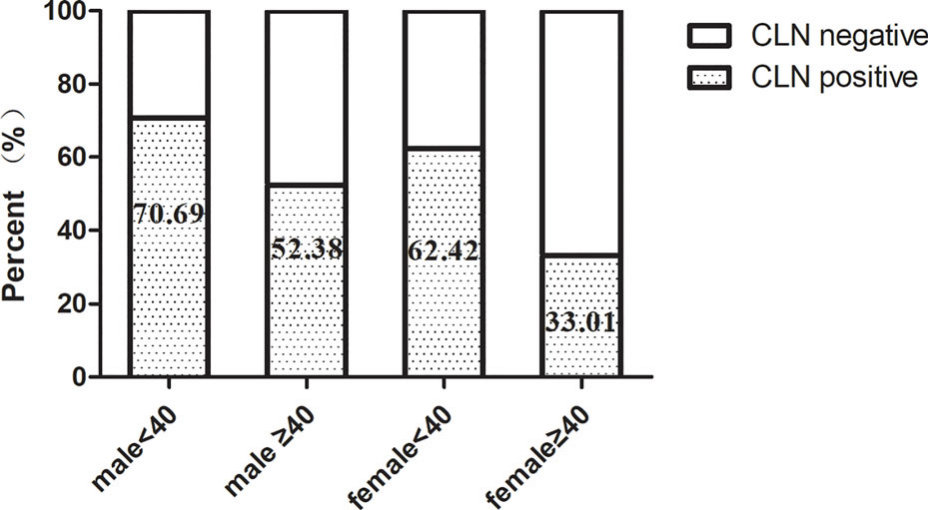
Central lymph node metastasis rate according to sex and age.

**Figure 2:**
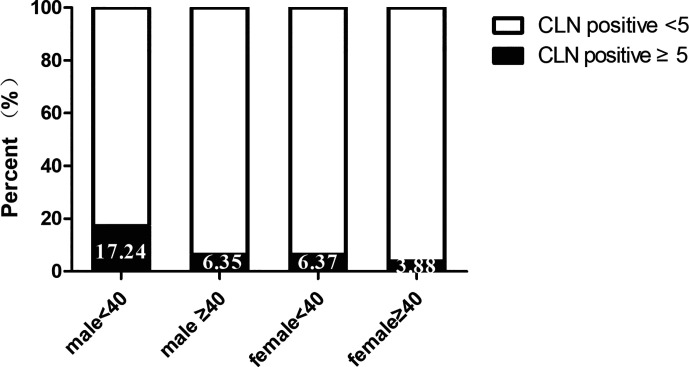
Central lymph node metastasis volume according to sex and age.

Univariate analysis was used to identify risks factors for CLNM in patients with PTMC. An increased risk of CLNM was associated with male sex, age <40 years, largest tumor diameter (LTD) ≥0.5 cm, and bilaterality (odds ratio [OR]=1.761 [95% confidence interval (CI), 1.113–2.787], p=0.016; OR=3.175 [95% CI, 2.145–4.698], p=0.00, OR=2.367 [95% CI, 1.43–3.919], p=0.001, OR=1.659 [95% CI, 0.943–2.92], p=0.079, respectively) (**Table 2**). Furthermore, multivariate analyses confirmed that male sex, age <40 years, LTD ≥0.5 cm, and bilaterality were independent risk factors for CLNM in patients with PTMC (OR=1.915 [95% CI, 1.225–2.994], p=0.004; OR=3.084 [95% CI, 2.095–4.54], p=0.00, OR=2.36 [95% CI, 1.44–3.868], p=0.001, OR=1.754 [95% CI, 1.06–2.902], p=0.029, respectively) (**Table 2**). As shown in **Figure 1**, male patients aged <40 years had a higher rate of CLNM than female patients aged over 40 years. In addition, the above-mentioned patients had larger volume of involved lymph node (number of CLNM ≥5) than others, as indicated in **Figure 2**.

**Table 2:** Univariate and multivariate analyses of risk factors for CLNM.

Variables	Univariate analyses		Multivariate analyses	
	Odds ratio (95% CI)	*P* value	Odds ratio (95% CI)	*P* value
Male sex	1.761 (1.113–2.787)	**0.016**	1.915 (1.225–2.994)	**0.004**
Age <40	3.175 (2.145–4.698)	**0.00**	3.084 (2.095–4.54)	**0.00**
LTD ≥0.5cm	2.367 (1.43–3.919)	**0.001**	2.36 (1.44–3.868)	**0.001**
Multifocal tumor	1.231 (0.618–2.451)	0.555		
Extrathyroidal invasion	1.313 (0.807–2.135)	0.273		
Vascular embolus	^a^	^a^		
Bilateral tumor	1.659 (0.943–2.92)	**0.079**	1.754 (1.06–2.902)	**0.029**
HT	0.764 (0.505–1.155)	0.201		

LTD, largest tumor diameter; HT, Hashimoto’s thyroiditis.

a The odds ratio was too high to report. Bold value indicates value with statistical significance.

### Univariate and Multivariate Analyses of Risk Factors for Lateral Lymph Node Metastases

A total of 9.1% patients experienced LLNM when they were first diagnosed with PTMC. Among patients with PTMC, patients with extrathyroidal invasion, presence of CLNM and CLNM ≥5 were associated with an increased risk of LLNM (OR=2.239 [95% CI 1.072–4.68], p=0.032; OR=8.625 [95% CI 2.196–33.869], p=0.002; and OR=3.749 [95% CI 1.484-9.472], p=0.005, respectively). Moreover, these three factors remained independent risk factors on multivariate analysis (OR=2.523 [95% CI 1.244–5.118], p=0.01; OR=11.362 [95% CI 3.383–38.16], p=0.00; OR=5.151 [95% CI 2.259-11.748], p=0.00, respectively) (**Table 3**).

**Table 3:** Univariate and multivariate analyses of risk factors for LLNM.

Variables	Univariate analyses		Multivariate analyses	
	Odds ratio (95% CI)	*P* value	Odds ratio (95% CI)	*P* value
Male sex	1.083 (0.493–2.376)	0.842		
Age <40	0.988 (0.482–2.024)	0.973		
LTD ≥0.5cm	2.336 (0.528–10.613)	0.261		
Multifocal tumor	1.505 (0.553–4.098)	0.424		
Extrathyroidal invasion	2.239 (1.072–4.68)	**0.032**	2.523 (1.244–5.118)	**0.01**
Vascular embolus	^a^	^a^		
Bilateral tumor	1.277 (0.511–3.19)	0.6		
HT	1.676 (0.796–3.529)	0.174		
Presence of CLNM	8.625 (2.196–33.869)	**0.002**	11.362 (3.383–38.16)	**0.00**
Number of CLNM ≥5	3.749 (1.484–9.472)	**0.005**	5.151 (2.259–11.748)	**0.00**
LN ratio	1.769 (0.447–6.997)	0.416		

LTD, largest tumor diameter; HT, Hashimoto’s thyroiditis.

a The odds ratio was too low to report. Bold value indicates value with statistical significance.

## Discussion

In our series, the number of female patients was triple those of males, which is consistent with the characteristics of thyroid malignancy of higher prevalence among women (1). Other studies have associated male sex and younger age with an increased rate of CLNM and disease recurrence, leading to poor prognosis (12–15). Accordingly, our data revealed that males and individuals aged under 40 years were more prone to CLNM in patients with PTMC. As compared with female PTMC patients aged 40 or more, large-volume CLNM (≥5), an independent predictor of LLNM, was more frequently observed in male patients aged under 40 years. Moreover, tumor size has been reported to be another essential predictor of CLNM and LLNM (14–16). Similarly, when we adopted tumor size of 5 mm as a threshold, we found that tumors with LTD ≥5 mm was significantly correlated with CLNM.

Bilateral tumors were observed in 18% PTMC cases in our cohort, which was in line with the description of previous studies (17, 18). In 2012, Wang et al. demonstrated that bilateral PTCs presented more advanced tumor stage and poorer prognosis than unilateral PTC (18). Later in 2016, the same conclusions were drawn by comparing the outcomes of bilateral and unilateral-multifocal PTCs, indicating that bilaterality, but not multifocality was associated with higher CLNM rates and shorter disease-free survival in PTC patients (19). Further analysis showed that bilateral PTC shared the same clonal origin, suggesting the presence of intrathyroidal metastasis, which may in part explain the aggressive features and high CLNM rates. However, different opinions have proposed, supporting the predictive role of multifocality for increased risk of CLNM and disease recurrence (20, 21). Our current study focused on PTMCs, and the results suggested that bilaterality had a stronger influence on CLNM than multifocality. Interestingly, investigating further, Wu et al. determined that for patients with unilateral multifocal PTMC, the sum of the diameter of all tumors ≥1.0 cm was an independent risk factor for CLNM (22).

HT refers to a common autoimmune thyroiditis that features lymphocytic infiltration and follicular destruction (23). The correlation between HT and TC is well-established yet debated. Some studies have indicated that PTC patients with concomitant HT exhibit aggressive behavior (14, 23), while others have reported a protective role of HT on cervical lymph node metastasis (24, 25). Further studies have revealed that the association between HT and TC could be age-related (26). Based on the findings of our study, no correlation between HT and CLNM or LLNM was identified in PTMC patients.

Considering the indolent course of PTMC, it is not recommended by the ATA guidelines to perform routine prophylactic central neck dissection in clinically node-negative PTMC patients (6). However, it is noteworthy that neck ultrasound has poor sensitivity in detecting metastases of the central compartment (11, 27). As for our cohort, among the 240 patients pathologically diagnosed as lymph node positive, only 21.7% were indicated by preoperative examination. Our result is consistent with the observation by Wu et al. that ultrasonography usually has sensitivity rates below 30% for CLNM (11). Also, it should be taken into consideration that CLND at re-operation would be complicated, and presents an increased risk of complications including laryngeal recurrent nerve injury and hypoparathyroidism (28).

Moreover, extrathyroidal invasion is indicative of locally advanced behavior, and tumors of any size presenting with extrathyroidal invasion are grouped into stage T4. Of note, our results showed that in over 20% of PTMC patients extrathyroidal invasion was confirmed on pathological examination, which resulted to be an independent risk factor for LLNM. Likewise, other studies have also described gross extrathyroidal invasion as a predictive factor for recurrence and poor outcomes, independently affecting disease-free survival and disease-specific survival (12, 14, 29).

In our series, 44 patients (9.1%) with PTMC presented with LLNM, with three patients exhibiting skip metastasis (i.e., the presence of LLNM without involving the central compartment). During subsequent analysis, we further divided patients into two groups according to the number and ratio of CLNM. Large-volume metastasis was defined by the presence of up to 5 metastatic CLN or a metastatic ratio of more than 50% and indicated adverse outcomes. Shou et al. observed significantly lower 5-year recurrence-free survival rates in cN0 PTMC patients with non-small-volume CLNM (metastatic lymph nodes >5 or ≥2 mm in size), indicating the negative role of large-volume metastasis (30). Likewise, our results demonstrated that both the presence of CLNM and CLNM ≥5 were strong indicators for LLNM in patients with PTMC. Therefore, for those underwent prophylactic central neck dissection and confirmed with positive CLNM or CLNM ≥5 at the pathological diagnosis, close follow-up may be warranted to screen for an increased risk of LLNM.

There are also several limitations to this study. First, this was a single-center retrospective study. Second, patients with CLNM were not differentiated by ipsilateral metastases or contralateral metastases due to the fact that the dissected lymph node samples were collectively defined as central lymph node in most cases; thus, further studies are required to address this issue. Finally, we calculated the incidence of temporary or permanent hypoparathyroidism but not the rates of laryngeal recurrent nerve injury given that the occurrence of hoarseness was not recorded in the medical records. However, based on our observations, no permanent vocal cord paralysis was reported for any of the patients evaluated.

## Conclusion

In the present study, we show that the incidence of CLNM in PTMC is not negligible, and is associated with several independent risk factors including male sex, age <40 years, largest tumor diameter ≥0.5 cm, and bilaterality. Furthermore, lateral lymph node metastasis was exhibited in some PTMC patients and was associated with tumor extrathyroidal invasion, presence of CLNM, and CLNM ≥5 lesions. Although active surveillance is recommended as an alternative approach by ATA guidelines for PTMCs with no locally advanced features, based on our findings we proposed that it should be considered more carefully for patients that undergo delayed surgical treatment as they may present with more aggressive features. Given the increased risks of complications during second operation, we believe that identification of potential risk factors for CLNM and LLNM would help tailor individual surgical interventions for patients with PTMC.

## Data Availability Statement

The raw data supporting the conclusions of this article will be made available by the authors, without undue reservation.

## Author Contributions

YH contributed to the conception and design of the study. WZ organized the database and acquired the data. YY performed the statistical analysis. WZ and YY wrote the first draft of the manuscript. YH critically revised the manuscript and supplemented the data for the final draft of the manuscript. All authors contributed to the article and approved the submitted version.

## Funding

This research was funded by the Medical Scientific Research Foundation of Guangdong Province of China (grant#A2020014).

## Conflict of Interest

The authors declare that the research was conducted in the absence of any commercial or financial relationships that could be construed as a potential conflict of interest.

## Abbreviations

ATA, American Thyroid Association; CI, Confidence interval; CLND, Central lymph node dissection; CLNM, Central lymph node metastases; FNA, Fine needle cytology aspiration; FTC, Follicular thyroid cancer; HT, Hashimoto’s thyroiditis; HTC, Hürthle cell thyroid cancer; LH, Lobo-isthmectomy; LLND, Lateral lymph node dissection; LLNM, Lateral lymph node metastases; LTD, Largest tumor diameter; OR, Odds ratio; PTC, Papillary thyroid cancer; PTH, Parathyroid hormone; PTMC, Papillary thyroid micro-carcinoma; TC, Thyroid cancer; TT, Total thyroidectomy.
